# Putative Membrane Receptors Contribute to Activation and Efficient Signaling of Mitogen-Activated Protein Kinase Cascades during Adaptation of Aspergillus fumigatus to Different Stressors and Carbon Sources

**DOI:** 10.1128/mSphere.00818-20

**Published:** 2020-09-16

**Authors:** Lilian Pereira Silva, Dean Frawley, Leandro José de Assis, Ciara Tierney, Alastair B. Fleming, Ozgur Bayram, Gustavo Henrique Goldman

**Affiliations:** a Faculdade de Ciências Farmacêuticas de Ribeirão Preto, Universidade de São Paulo, São Paulo, Brazil; b Biology Department, Maynooth University, Maynooth, Co. Kildare, Ireland; c Department of Microbiology, School of Genetics and Microbiology, Moyne Institute of Preventive Medicine, Trinity College Dublin, Dublin, Ireland; University of Georgia

**Keywords:** *Aspergillus fumigatus*, putative receptors, osmotic and cell wall stresses, high-osmolarity glycerol (HOG), caspofungin

## Abstract

The high-osmolarity glycerol (HOG) response pathway is a multifunctional signal transduction pathway that specifically transmits ambient osmotic signals. Saccharomyces cerevisiae Hog1p has two upstream signaling branches, the sensor histidine kinase Sln1p and the receptor Sho1p. The Sho1p branch includes two other proteins, the Msb2p mucin and Opy2p. Aspergillus fumigatus is the leading cause of pulmonary fungal diseases. Here, we investigated the roles played by A. fumigatus SlnA^Sln1p^, ShoA^Sho1p^, MsbA^Msb2p^, and OpyA^Opy2p^ putative homologues during the activation of the mitogen-activated protein kinase (MAPK) HOG pathway. The *shoA*, *msbA*, and *opyA* singly and doubly null mutants are important for the cell wall integrity (CWI) pathway, oxidative stress, and virulence as assessed by a Galleria mellonella model. Genetic interactions of ShoA, MsbA, and OpyA are also important for proper activation of the SakA^Hog1p^ and MpkA^Slt2^ cascade and the response to osmotic and cell wall stresses. Comparative label-free quantitative proteomics analysis of the singly null mutants with the wild-type strain upon caspofungin exposure indicates that the absence of ShoA, MsbA, and OpyA affects the osmotic stress response, carbohydrate metabolism, and protein degradation. The putative receptor mutants showed altered trehalose and glycogen accumulation, suggesting a role for ShoA, MsbA, and OpyA in sugar storage. Protein kinase A activity was also decreased in these mutants. We also observed genetic interactions between SlnA, ShoA, MsbA, and OpyA, suggesting that both branches are important for activation of the HOG/CWI pathways. Our results help in the understanding of the activation and modulation of the HOG and CWI pathways in this important fungal pathogen.

**IMPORTANCE** Aspergillus fumigatus is an important human-pathogenic fungal species that is responsible for a high incidence of infections in immunocompromised individuals. A. fumigatus high-osmolarity glycerol (HOG) and cell wall integrity pathways are important for the adaptation to different forms of environmental adversity such as osmotic and oxidative stresses, nutrient limitations, high temperatures, and other chemical and mechanical stresses that may be produced by the host immune system and antifungal drugs. Little is known about how these pathways are activated in this fungal pathogen. Here, we characterize four A. fumigatus putative homologues that are important for the activation of the yeast HOG pathway. A. fumigatus SlnA^Sln1p^, ShoA^Sho1p^, MsbA^Msb2p^, and OpyA^Opy2p^ are genetically interacting and are essential for the activation of the HOG and cell wall integrity pathways. Our results contribute to the understanding of A. fumigatus adaptation to the host environment.

## Abstract (precis)

Aspergillus fumigatus is an important human-pathogenic fungal species that is responsible for a high incidence of infections in immunocompromised individuals. A. fumigatus high-osmolarity glycerol (HOG) and cell wall integrity pathways are important for the adaptation to different forms of environmental adversity such as osmotic and oxidative stresses, nutrient limitations, high temperatures, and other chemical and mechanical stresses that may be produced by the host immune system and antifungal drugs. Little is known about how these pathways are activated in this fungal pathogen. Here, we characterize four A. fumigatus putative homologues that are important for the activation of the yeast HOG pathway. A. fumigatus SlnA^Sln1p^, ShoA^Sho1p^, MsbA^Msb2p^, and OpyA^Opy2p^ are genetically interacting and are essential for the activation of the HOG and cell wall integrity pathways. Our results contribute to the understanding of A. fumigatus adaptation to the host environment.

## INTRODUCTION

Aspergillus fumigatus causes aspergillosis, which includes chronic pulmonary aspergillosis (CPA), allergic bronchopulmonary aspergillosis (ABPA), and invasive pulmonary aspergillosis (IPA) (1). IPA has a mortality rate of 50% to 75% when treated, affecting primarily immunocompromised individuals, patients with cancer or hematological neoplasms, or patients undergoing chemotherapy (1–3). A. fumigatus is able to adapt to different forms of environmental adversity such as osmotic and oxidative stresses, nutrient limitations, high temperatures, and other chemical and mechanical stresses, some of which may be produced by the host immune system and antifungal drugs (4). The survival capacity of A. fumigatus under different stress conditions depends on its response and adaptation mechanisms (4).

**FIG 1:**
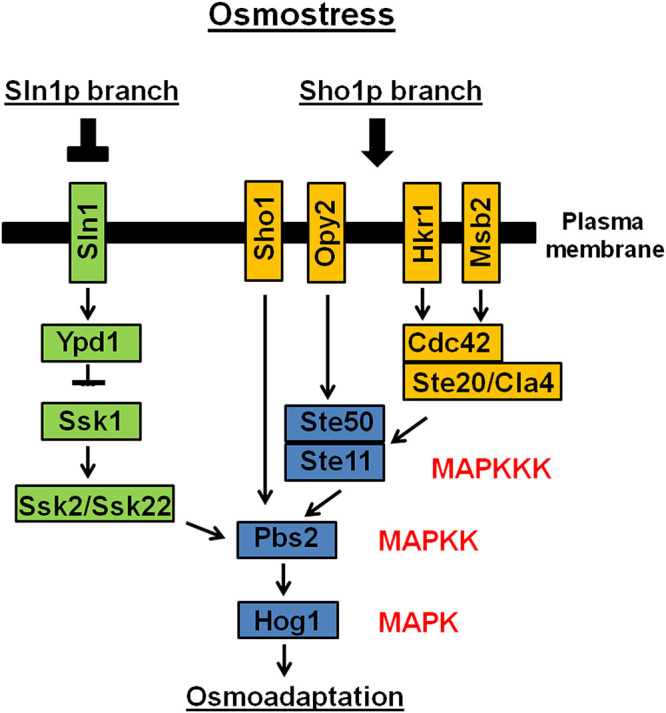
The two branches of Snl1 and Sho1 which are responsible for the activation of the S. cerevisiae high-osmolarity glycerol (HOG) pathway. (Adapted from reference 43).

The high-osmolarity glycerol (HOG) response pathway is a multifunctional signal transduction pathway that specifically transmits ambient osmotic signals (5). The HOG pathway is activated by mitogen-activated protein kinases (MAPKs) during adaptation to environmental stress and morphology regulation (5). The MAPK cascades consist of three kinases, MAPK, MAPK kinase (MAPKK), and MAPKK kinase (MAPKKK), which are highly conserved in fungi (6–8). After the environmental stimulus, the MAPK cascade is activated by sequential phosphorylation, resulting in the activation of transcription factors and expression of target genes that assist in the adaptation to a given condition (5, 9). In Saccharomyces cerevisiae, Hog1p has the following two upstream signaling branches (9–11) (Fig. 1): (i) the Sho1p (synthetic high osmolarity) membrane protein, containing four N-terminal transmembrane domains (TM) and one C-terminal domain (SH3) (9, 11, 12), and (ii) Sln1p (synthetic lethal of N-end rule), a transmembrane histidine phosphotransfer kinase and osmosensor with an intracellular kinase domain forming a phosphorelay system similar to bacterial two-component regulators (9, 11). In yeast, Sho1p is responsible for transmitting signals of osmotic stress by sequentially activating the MAPKKs Pbs2p and Hog1p. In addition, the Sho1p branch includes two other TM proteins, an Msb2p (multicopy suppressor of a budding defect) mucin and an Opy2p (overproduction-induced pheromone-resistant yeast) type 1 TM ancestral protein (13, 14). Under conditions of hyperosmotic stress, these TM proteins form a complex that is composed of two GTPases (Cdc42p and Cdc24p) and three kinases (Ste11p, Ste50p, and Ste20p) and that activates the Hog1p pathway (Fig. 1) (15–17).

The Sln1 branch of the yeast HOG pathway is an example of a two-component system (11, 18, 19). The typical organization of a two-component system consists of the following components: (i) a sensor histidine kinase (SHK) that contains an input (or sensor) domain, an HK catalytic domain, and a histidine autophosphorylation site and (ii) a response regulator (RR) that contains a receiver (REC) domain and an output (or effector) domain (20). The sensor domain is modified by a stimulus, a histidine close to the HK domain is phosphorylated (or dephosphorylated), and this phosphoryl group is transferred to the REC domain of the RR (20). Yeast has three REC proteins (Sln1, Ssk1, and Skn7), one SHK (Sln1), and one HPt (Ypd1) (Fig. 1). S. cerevisiae Sln1 is responsible for the coordination of two distinct signaling pathways: the Sln1-Ypd1-Ssk1 phosphorelay pathway, which is important for the regulation of hyperosmolarity responses, and the Sln1-Ypd1-Skn7 pathway, which is important for the regulation of hypo-osmolarity responses (11).

In A. fumigatus, ShoA, the putative homologue of S. cerevisiae Sho1p, has been shown to be important for fungal morphology and oxidative stress; however, a thorough investigation of ShoA function has not been performed (21). The A. fumigatus Sln1p homologue has been previously characterized (22). The Δ*tcsB^slnA/sln1p^* mutant was partially sensitive to SDS, and Western blot analysis of both the corresponding wild-type strain and the Δ*tcsB^slnA/sln1p^* mutant showed that when stressed with hydrogen peroxide, phosphorylation of Hog1p still occurred in the mutant (22). The A. fumigatus Msb2p mucin homologue, MsbA, has been shown to be important for fungal development, biofilm formation, and cell wall integrity (CWI) (23). The function of the TM Opy2p putative homologue in A. fumigatus (here named OpyA) has not been elucidated, and the interactions of the ShoA branch with the MsbA and OpyA and SlnA^Sln1p^ branches during HOG MAPK pathway activation are undefined.

Four MAPKs have been identified in A. fumigatus: (i) MpkA, which is important for the CWI pathway and oxidative stress (22, 24, 25); (ii) MpkB, which regulates the pheromone response/filamentous growth pathway, conidiation, and dihydroxynaphthalene (DHN) melanin production (26, 27); and (iii) SakA and (iv) MpkC, which are paralogues that regulate the HOG pathway (28). MpkC and SakA interact physically and play roles in caspofungin tolerance and carbon source utilization, respectively (24, 29–33). We do not have very much information about the upstream mechanisms by which MpkC and SakA are activated and execute their signaling functions.

Here, we investigated in more detail the roles played by both the Sln1 and Sho1 A. fumigatus putative branches during the activation of the MAPK HOG pathway. Our results strongly indicate that these four proteins actively collaborate to promote activation of osmotic stress responses and the cell wall integrity pathway (CWI) MpkA MAPK in A. fumigatus.

## RESULTS

### The Sho1 branch: functional characterization of three A. fumigatus genes encoding putative receptors.

**FIG 2:**
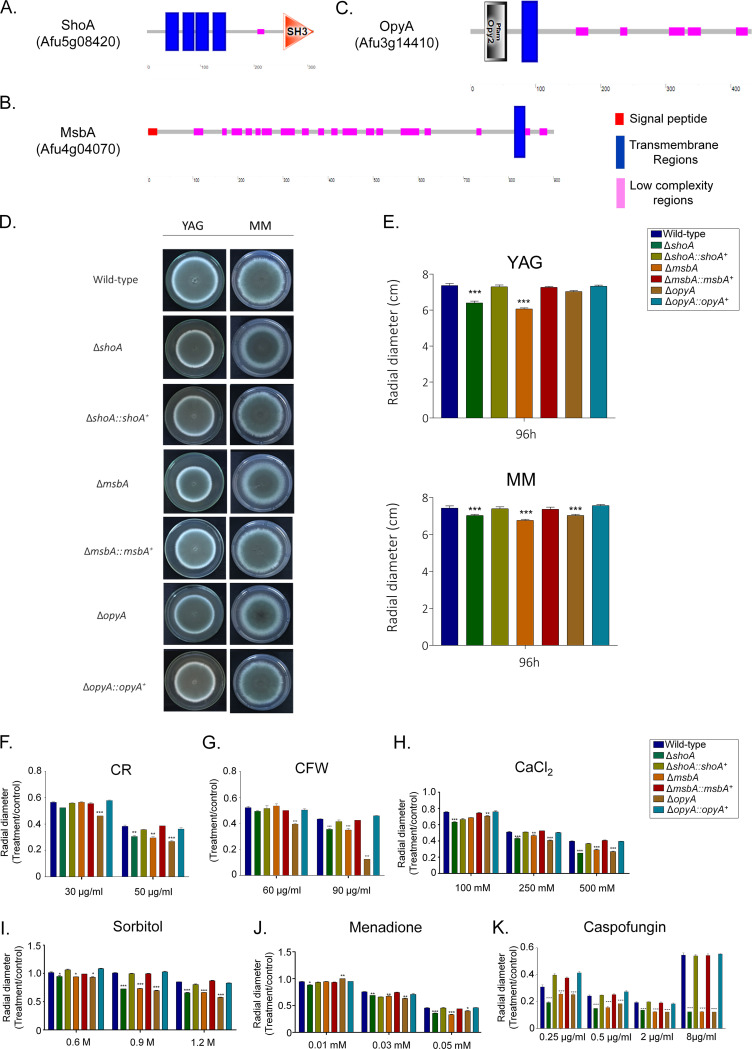
A. fumigatus ShoA, MsbA, and OpyA. (A to C) Protein organization of A. fumigatus ShoA (A), MsbA (B), and OpyA (C). (D to J) Growth phenotypes of the wild-type strain and the Δ*shoA*, Δ*msbA*, Δ*opyA*, Δ*shoA*::*shoA^+^*, Δ*msbA*::*msbA^+^*, and Δ*opyA*::*opyA^+^* mutants. The strains were grown for 4 days at 37°C (D) on MM or YAG (E), MM plus Congo red (CR) (F), MM plus calcofluor white (CFW) (G), MM plus CaCl_2_ (H), MM plus sorbitol (I), MM plus menadione (J), and MM plus caspofungin (K), and their radial growth was quantified. The results shown represent the means of measurements of the diameter of 3 colonies for each strain ± standard deviation. The data were analyzed (Prism, GraphPad) using two-way ANOVA followed by Bonferroni posttests. The levels of significance compared to the wild-type results are indicated as follows: *, *P* < 0.1; **, *P* < 0.01; ***, *P* < 0.001.

A. fumigatus ShoA and MsbA homologues were previously identified (21, 23). We validated their organization and identified A. fumigatus putative homologues of S. cerevisiae Opy2p. (i) ShoA^Sho1p^ (A. fumigatus B_055960 [AFUB_055960]) has 311 amino acids and 30% identity and 48% similarity with Sho1p (E value = 4e−39). It has four transmembrane regions and a Src homology 3 (SH3) domain (Interpro IPR001452) that binds to target proteins through sequences containing proline and hydrophobic amino acids (Fig. 2A). (ii) MsbA^Msb2p^ (AFUB_098950) has 901 amino acids and 34% identity and 55% similarity with Msb2p (E value = 8e−12). It has a putative signal peptide (from amino acids 1 to 21) and a single transmembrane region at the C terminus (Fig. 2B). (iii) OpyA^Opy2p^ (AFUB_034820) has 432 amino acids and very low identity with Opy2p that is restricted to the Opy2 domain that acts as a membrane anchor in the HOG signaling pathway (Interpro IPR018571, from amino acids 22 to 56) and a single transmembrane region located at the N terminus (Fig. 2C).

Null mutant strains for *shoA*, *msbA*, and *opyA* were constructed and were functionally complemented by homologous integration (Fig. 2D and E; see also Fig. S1 at https://figshare.com/articles/Membrane_receptors_contribute_to_activation_and_efficient_signaling_of_Mitogen-Activated_Protein_Kinase_cascades_during_adaptation_of_Aspergillus_fumigatus_to_different_stressors_and_carbon_sources/12402125). They had about 15% reduced growth compared to wild-type strain in YAG (except for the Δ*opyA* mutant, which showed the same radial diameter as the wild-type strain) and minimal medium (MM) (Fig. 2D and E). They were also phenotypically characterized under different stress conditions such as cell wall damage and osmotic and oxidative stresses (Fig. 2F to K). All three mutants were more sensitive to Congo red (CR), calcofluor white (CFW), CaCl_2_, sorbitol, and menadione than the wild-type and corresponding complementing strains (Fig. 2F to J). All three mutants were more sensitive to caspofungin, an echinocandin that noncompetitively inhibits β-1,3-glucan synthase, impairing fungal cell wall polysaccharide biosynthesis and integrity (Fig. 2K) (24, 34). Caspofungin paradoxical effect (CPE) is described as a phenomenon where high caspofungin concentrations restore the expected inhibition of A. fumigatus growth (35). All three mutants lost the CPE (Fig. 2K). A summary of the phenotypes is shown in Table 1. These results indicate that ShoA, MsbA, and OpyA are important for the activity of the CWI pathway and for responses to osmotic and oxidative stresses.

**TABLE 1:**
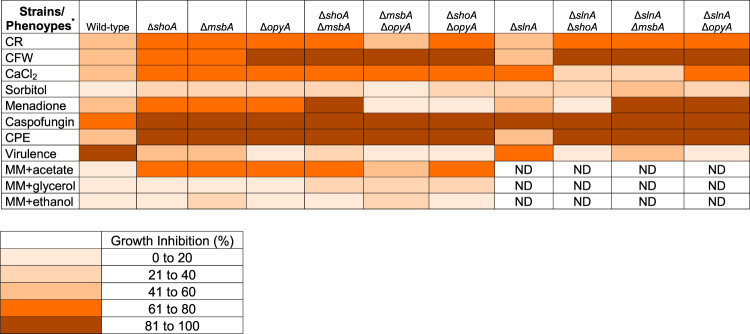
Summary of the growth inhibition of the wild-type strain and *slnA*, *shoA*, *msbA*, and *opyA* single and double mutants grown under different stressing conditions

*CR, Congo red (50 μg/ml); CFW, calcofluor white (90 μg/ml); CaCl_2_ (500 mM); Sorbitol 1.2 M; Menadione 0.05 M; Caspofungin (2 μg/ml); CPE, Caspofungin Paradoxical Effect (8 μg/ml); Virulence, virulence in Galleria mellonella; MM+acetate 1%; MM+glycerol 1%; MM+ethanol 1%; ND, not determined.

### ShoA, MsbA, and OpyA null mutants have genetic interactions.

**FIG 3:**
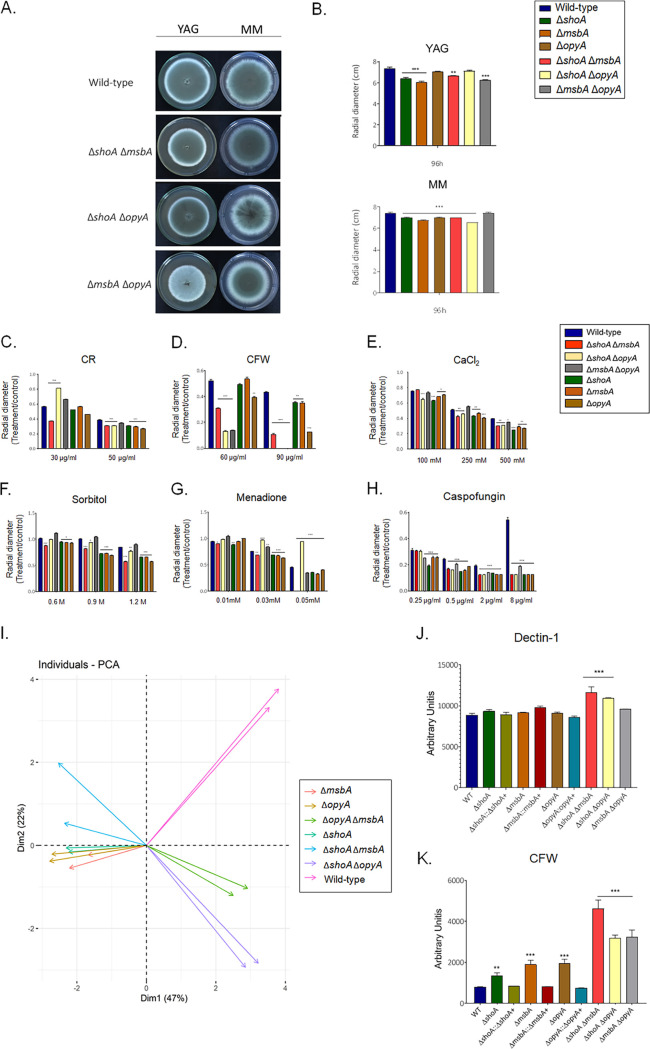
A. fumigatus double null mutants indicate genetic interactions among Δ*shoA*, Δ*msbA*, and Δ*opyA.* Growth phenotypes of the wild-type strain and the Δ*shoA*, Δ*msbA*, Δ*opyA*, Δ*msbA* Δ*opyA*, Δ*shoA* Δ*msbA*, and Δ*shoA* Δ*opyA* mutants were determined. (A to H) The strains were grown for 4 days at 37°C (A) on MM or YAG (B) or on MM plus Congo red (CR) (C), MM plus calcofluor white (CFW) (D), MM plus CaCl_2_ (E), MM plus sorbitol (F), and MM plus menadione (G), and (H) MM plus caspofungin, and their radial growth was quantified. The results shown represent the means of measurements of the diameter of 3 colonies for each strain ± standard deviation. The data were analyzed (Prism, GraphPad) using two-way ANOVA followed by Bonferroni posttests. The levels of significance compared to the wild-type results are indicated as follows: *, *P* < 0.1; **, *P* < 0.01; ***, *P* < 0.001. (I) PCA of the results shown in panels A to E. (J and K) Detection of β-1,3−glucan (Dectin-1; J) and chitin (CFW; K) exposed on the cell surface was performed. WT, wild type. Experiments were performed in triplicate, and the results are displayed as mean values with standard errors (two-way ANOVA followed by Tukey’s *P < *0.05).

To investigate possible genetic interactions between these putative receptors, we constructed Δ*shoA* Δ*msbA*, Δ*shoA* Δ*opyA*, and Δ*msbA* Δ*opyA* double mutants (Fig. 3A and B). The double mutants showed about 15% reduced growth compared to the wild-type strain in YAG and MM (Fig. 3A and B). All of the double mutants showed levels of susceptibility to CR 50 μg/ml similar to those seen with the corresponding single mutants (Fig. 3C), suggesting that *shoA*, *msbA*, and *opyA* are functioning in the same pathway to repair and/or process cell wall damage caused by CR (Tables 1 and 2). In contrast, all the double mutants showed increased susceptibility to CFW 90 μg/ml in comparison to the corresponding single mutants, suggesting an additive interaction (Fig. 3D; see also Tables 1 and 2). The double mutants were also as sensitive to 500 mM CaCl_2_ as the corresponding single mutants, suggesting again that *shoA*, *msbA*, and *opyA* function in the same pathway for calcium signaling (Fig. 3E). The double mutants, except the Δ*msbA* Δ*opyA* mutant, were as sensitive to osmotic stress caused by 1.2 M sorbitol as the corresponding single mutants (Fig. 3F; see also Tables 1 and 2), suggesting once more that *shoA* and *msbA* and *shoA* and *opyA* function in the same pathway for osmotic stress signaling. However, the Δ*msbA* Δ*opyA* mutant was more resistant to osmotic stress (Fig. 3F; see also Tables 1 and 2), suggesting that *msbA* and *opyA* do not genetically interact. The Δ*shoA* Δ*msbA* mutant was more sensitive to oxidative stress caused by 0.05 mM menadione than the corresponding single mutants, suggesting an additive interaction (Fig. 3G; see also Tables 1 and 2) while the Δ*msbA* Δ*opyA* and Δ*shoA* Δ*opyA* mutants were less sensitive than the corresponding single mutants (Fig. 3G; see also Tables 1 and 2), suggesting suppression interactions. All the double mutants were as sensitive to lower caspofungin and CPE concentrations as the single mutants (Fig. 3H; see also Tables 1 and 2), suggesting that they function in the same pathway for caspofungin signaling.

**TABLE 2:**
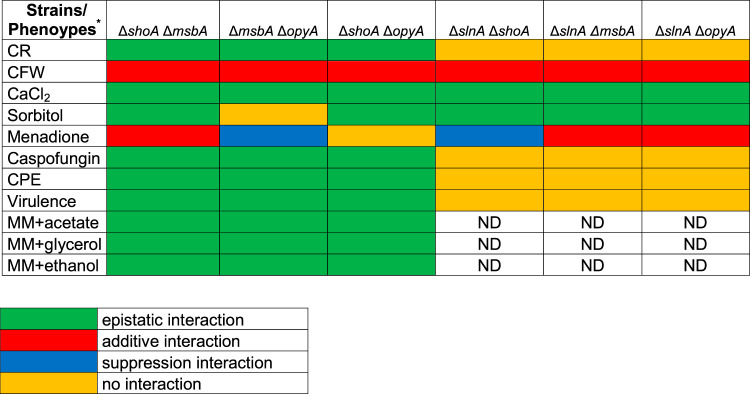
Summary of the genetic interactions between *slnA*, *shoA*, and *msbA* single mutants grown under different stressing conditions

* CR, Congo red (50 μg/ml); CFW, calcofluor white (90 μg/ml); CaCl_2_ (500 mM); Sorbitol 1.2 M; Menadione 0.05 M; Caspofungin (2 μg/ml); CPE, Caspofungin Paradoxical Effect (8 μg/ml); Virulence, virulence in Galleria mellonella; MM+acetate 1%; MM+glycerol 1%; MM+ethanol 1%; ND, not determined.

Considering the paucity of genetic transformation markers for A. fumigatus, we did not complement the double mutants. However, we tested few of the phenotypes described above for two independent candidates of the double mutants (see Fig. S2). They showed exactly the same phenotypes, strongly indicating that there were no additional mutations that were introduced into these double mutants during the transformation process and that could be responsible for the observed phenotypes (see Fig. S2).

The differences between the mutant and wild-type strains were analyzed through a principal-component analysis (PCA) (Fig. 3I). This analysis was performed with the set of all data obtained through measurement of the radial growth of each strain under different stress conditions. In the spatial distribution of the graph, we can see that the single Δ*shoA*, Δ*msbA*, and Δ*opyA* mutants are grouped, indicating that the single mutants showed similar phenotypes after growth under different stress conditions (CR, CFW, menadione, and sorbitol). These data show that ShoA, MsbA, and OpyA have similar and/or redundant functions.

Looking at the result of the PCA for the double mutants, we can see that the Δ*msbA* Δ*opyA* and Δ*shoA* Δ*opyA* double mutants are similar and also grouped apart from the wild-type and the single mutants (Fig. 3H). This suggests that ShoA can fulfill some of the functions performed by MsbA in the Δ*msbA* Δ*opyA* double mutant. Similarly, MsbA can fulfill some of the functions of ShoA in the Δ*shoA* Δ*opyA* double mutant. As a consequence, when we subjected the double mutants to stress conditions, we observed that the Δ*shoA* Δ*opyA* and Δ*msbA* Δ*opyA* double mutants showed the same behavior.

Finally, we observed that the Δ*shoA* Δ*msbA* double mutant was different from the wild-type strain, the single mutants, and the other two double mutants (the Δ*shoA* Δ*opyA* and Δ*msbA* Δ*opyA* mutants). This suggests that ShoA and MsbA have unique functions during signaling caused by stressful conditions. A summary of the phenotypes of and the genetic interactions between the single mutants is shown in Tables 1 and 2. The data set shows that these putative TM proteins can have redundant as well as unique functions during the activation of the signaling pathways in A. fumigatus.

Next, we investigated the cell wall organization using the exposure of different polysaccharides on the cell surface. All the single and double mutants showed a germling length comparable to that seen with the wild-type strains after 16 h of growth at 37°C without shaking. Dectin-1 binding results revealed increased exposure of β-glucans only in the Δ*shoA* Δ*opyA* and Δ*shoA* Δ*msbA* cell walls compared with the wild-type strain and all the other mutants (Fig. 3I). However, CFW staining showed that the Δ*shoA*, Δ*opyA*, and Δ*msbA* mutants exhibited an increase in chitin exposure in comparison to the wild-type and complemented strains (Fig. 3J). The double mutants once more showed a synergistic interaction because their level of chitin exposure was much higher than that of the corresponding single mutants (Fig. 3J).

Taken together, these results strongly indicate a genetic interaction among ShoA, MsbA, and OpyA in response to several stressing conditions that affect the cell wall structure and organization and responses to oxidative and osmotic stresses.

### ShoA, MsbA, and OpyA null mutants influence SakA and MpkA phosphorylation.

**FIG 4:**
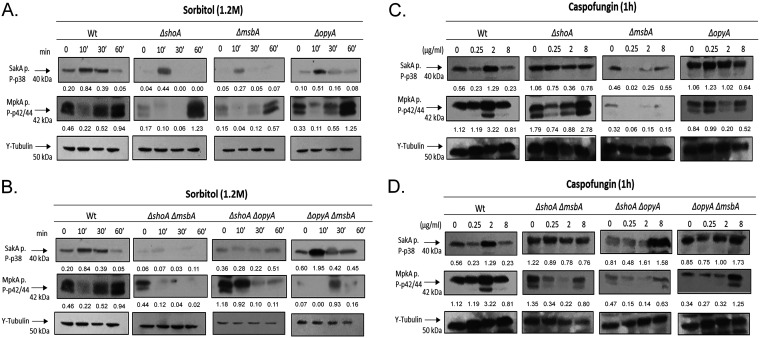
A. fumigatus ShoA, MsbA, and OpyA are important for SakA and MpkA phosphorylation. Western blotting assays of SakA and MpkA phosphorylation in the wild-type strain and single mutants (A and C) or in the wild-type strain and double mutants (B and D) in response to 1.2 M sorbitol for different periods of time (A and B) and in response to caspofungin 0.25, 2.0, or 8.0 μg/ml for 1 h (C and D) were performed. Anti-P-p38 SakA and anti-44/42 MpkA antibodies were used to detect the phosphorylation of SakA and MpkA, respectively, while anti-γ-tubulin was used to detect γ-tubulin. Signal intensities were quantified using ImageJ software, and ratios of (P)-SakA to γ-tubulin or (P)-MpkA to γ-tubulin were calculated.

Subsequently, we investigated the impact of the deletion of the three putative receptors on the phosphorylation of MAP kinases SakA and MpkA in the presence of higher osmotic concentrations (1.2 M sorbitol) or different caspofungin concentrations (Fig. 4; for an additional repetition of the Western blot assays, see Fig. S3). These Western blot assays were semiquantitative experiments, and the results were somewhat variable. We focus on the trends that were common to the two independent experiments, though the precise fold changes might have differed between the experiments. The wild-type strain showed increased (about 4-fold) SakA phosphorylation when exposed for 10 min to 1.2 M sorbitol (Fig. 4A). In contrast, the Δ*shoA*, Δ*msbA*, and Δ*opyA* single mutants showed higher levels of SakA phosphorylation than the wild-type strain, with 11-fold, 5.4-fold, and 5-fold phosphorylation increases at 10 min, respectively (Fig. 4A). The Δ*shoA* Δ*msbA* and Δ*shoA* Δ*opyA* double mutants had a synergistic interaction, since both mutants showed much lower SakA phosphorylation than the corresponding single mutants whereas the Δ*opyA* Δ*msbA* double mutant showed about 3-fold SakA phosphorylation at 10 min (Fig. 4B).

The wild-type strain exhibited increased (about 2-fold) MpkA phosphorylation when exposed to 1.2 M sorbitol for 60 min (Fig. 4A). All three single mutants show higher induction of MpkA phosphorylation levels than the wild-type strain at 60 min (Fig. 4A). The double mutants Δ*shoA* Δ*msbA* and Δ*shoA* Δ*opyA* showed lower MpkA phosphorylation levels than the corresponding single mutants, while the Δ*opyA* Δ*msbA* double mutant showed about 13-fold- and 2-fold-increased MpkA phosphorylation at 30 and 60 min exposure to sorbitol, respectively, compared to the control without sorbitol (Fig. 4B).

When exposed to 2 μg/ml of caspofungin, the wild-type strain showed 2-fold and 2.8-fold more phosphorylation of SakA and MpkA, respectively (Fig. 4C). The Δ*shoA*, Δ*msbA*, and Δ*opyA* mutants showed no induction of either SakA or MpkA at 2 μg/ml of caspofungin, but the Δ*shoA* mutant showed about 1.6-fold induction of MpkA phosphorylation at 8 μg/ml of caspofungin (Fig. 4C). The Δ*shoA* Δ*msbA* double mutant showed no increase in SakA and MpkA phosphorylation, while the Δ*shoA* Δ*opyA* mutant showed about 2-fold SakA phosphorylation at 2 and 8 μg/ml of caspofungin but no MpkA phosphorylation at any caspofungin concentration (Fig. 4D). The Δ*opyA* Δ*msbA* double mutant showed no SakA phosphorylation in the presence of caspofungin but about 3.7-fold MpkA phosphorylation at 8 μg/ml of caspofungin (Fig. 4D).

Taken together, these results strongly indicate that ShoA, MsbA, and OpyA can affect the levels of MpkA and SakA phosphorylation when A. fumigatus is exposed to osmotic and cell wall stresses.

**FIG 5:**
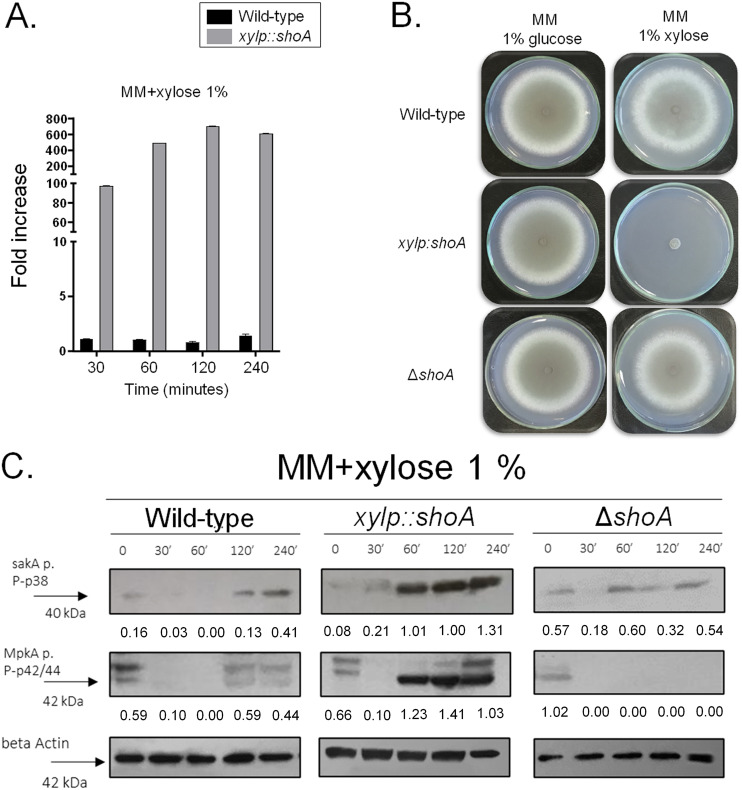
A. fumigatus *shoA* overexpression inhibits growth. (A) The wild-type and *xylp*::*shoA* strains were grown in MM plus 1% glucose for 16 h at 37°C and transferred to MM plus 1% xylose for 30 to 120 min at 37°C. The results shown represent the means of measurements of the diameter of 3 colonies for each strain ± standard deviation. Gene expression was normalized using *tubA* (Afu1g10910). Standard deviations present averages of results from three independent biological repetitions (each performed with 2 technical repetitions). (B) The wild-type, *xylp*::*shoA*, and Δ*shoA* strains were grown either on MM plus 1% glucose or on MM plus 1% xylose for 5 days at 37°C. (C) The wild-type, *xylp*::*shoA*, and Δ*shoA* strains were grown in MM plus 1% glucose for 16 h at 37°C and transferred to MM plus 1% xylose for 30 to 120 min at 37°C. Western blotting assays of SakA and MpkA phosphorylation were performed. Anti-P-p38 SakA and anti-44/42 MpkA antibodies were used to detect the phosphorylation of SakA and MpkA, respectively.

After several attempts, we were unable to construct a Δ*shoA* Δ*msbA* Δ*opyA* triple mutant. We therefore attempted to construct a conditional *xylp*::*shoA* Δ*msbA* Δ*opyA* mutant strain by replacing the *shoA* endogenous promoter with the *xylp* promoter from Penicillium chrysogenum, which was induced by xylose and was repressed by glucose (36) in the Δ*msbA* Δ*opyA* strain. However, when this approach failed, we successfully replaced the *shoA* endogenous promoter with the *xylp* promoter in the wild-type strain (see Fig. S1). The *xylp*::*shoA* strain showed 100-fold and 600-fold increases in the levels of *shoA* transcripts when exposed for 30 and 120 min to xylose 1%, respectively, compared to the wild-type strain (Fig. 5A). *shoA* overexpression caused an almost complete reduction of growth rate in the *xylp*::*shoA* strain compared to the wild-type strain grown on MM plus 1% xylose (Fig. 5B). Overexpression of *shoA* also resulted in increased SakA phosphorylation (2.6-fold to 16-fold) and MpkA phosphorylation (about 2-fold) in comparison to the control (strain *xylp*::*shoA* grown in 1% glucose) (Fig. 5C). The levels of SakA and MpkA phosphorylation in the wild-type strain grown in xylose showed about a 2.5-fold increase and no increase, respectively, compared to the wild-type strain grown in glucose 1% (Fig. 5C). The levels of SakA and MpkA phosphorylation showed no increase in the Δ*shoA* mutant grown in xylose 1% compared to glucose 1% (Fig. 5C).

Taken together, these results strongly indicate that ShoA, MsbA, and OpyA genetic interactions are important for proper activation of the SakA and MpkA cascade and the response to osmotic and cell wall stresses.

### ShoA, MsbA, and OpyA are important for A. fumigatus virulence in Galleria mellonella.

**FIG 6:**
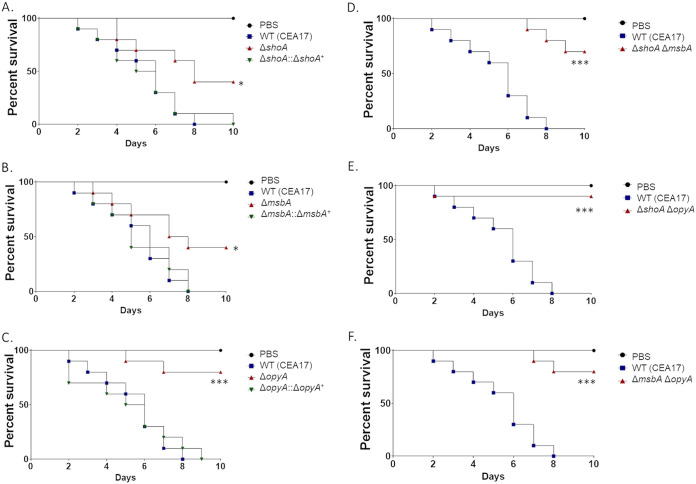
A. fumigatus *shoA*, *msbA*, and *opyA* mutants are important for virulence in Galleria mellonella. Cumulative survival rates of wild-type and *shoA* (A) *msbA* (B), and *opyA* (C) single mutant strains and wild-type and Δ*shoA* Δ*msba* (D) Δ*shoA* Δ*opyA* (E), and Δ*msbA* Δ*opyA* (F) double mutant strains in the model moth Galleria mellonella are shown. Infection of larvae was carried out via inoculation of 10^6^ conidia. For inoculations, 10 larvae were infected per wild-type or null mutant strain. The levels of significance compared to the wild-type results are indicated as follows: *, *P* < 0.1; **, *P* < 0.01; ***, *P* < 0.001. Phosphate buffered saline (PBS) was used as a negative control.

G. mellonella larvae were used to evaluate the importance of ShoA, MsbA, and OpyA in regulating A. fumigatus pathogenicity (Fig. 6). In the G. mellonella model, infection by the wild‐type strain resulted in 100% mortality 8 days postinfection (Fig. 6). However, the Δ*shoA* and Δ*msbA* mutants showed only 60% mortality rates 10 days postinfection, which was statistically significantly different from the results seen with the wild-type strain according to Mantel-Cox and Gehan-Breslow-Wilcoxon tests (*P* < 0.001; Fig. 6A and B). The Δ*opyA* mutant caused 20% mortality 10 days postinfection, which was not statistically significantly different from the results seen with the phosphate-buffered saline (PBS) control according to the MantelCox and Gehan-Breslow-Wilcoxon tests (*P* < 0.001; Fig. 6C). Interestingly, infection by all three double mutants resulted in 10% to 30% mortality rates 10 days postinfection, which was not statistically significantly different from the phosphate buffer saline (PBS) control results according to the Mantel-Cox and Gehan-Breslow-Wilcoxon tests (*P* < 0.001; Fig. 6D to F), suggesting that *shoA*, *msbA*, and *opyA* function in the same pathway for the establishment of virulence in A. fumigatus (Tables 1 and 2).

Taken together, these results clearly demonstrate that ShoA, MsbA, and OpyA play an important role in A. fumigatus virulence.

### Proteomic analysis reveals that ShoA, MsbA, and OpyA contribute to the metabolic modulation of A. fumigatus.

**FIG 7:**
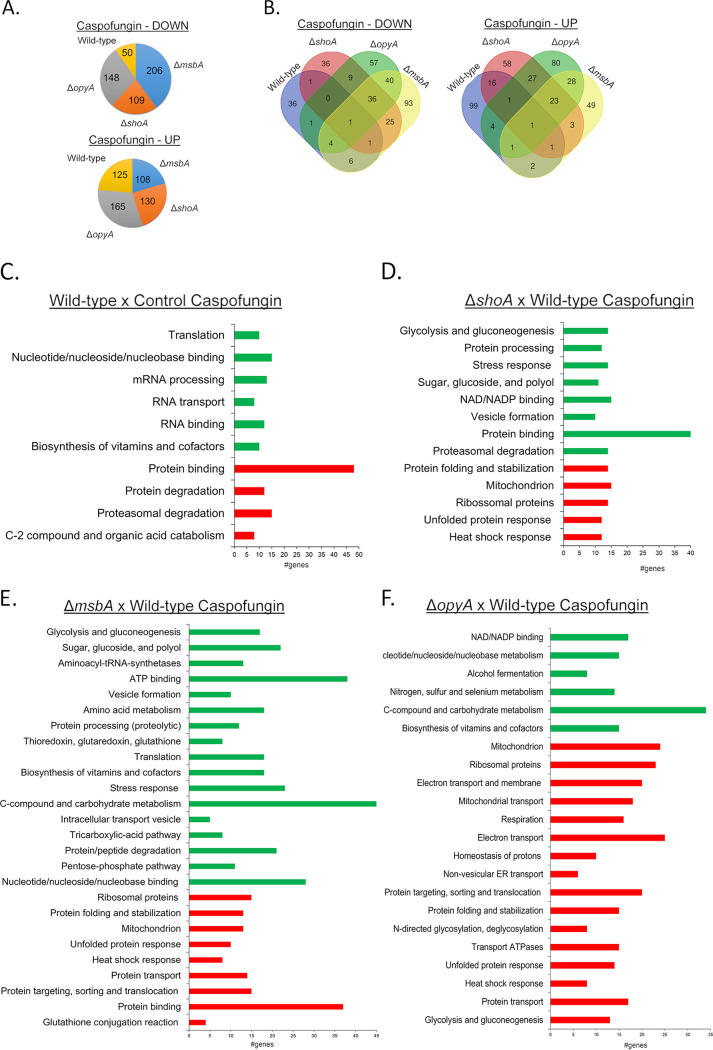
Proteomic analysis of the A. fumigatus wild-type strain and *shoA*, *msbA*, and *opyA* null mutants exposed to caspofungin. (A) Number of proteins with increased (UP) and decreased (DOWN) abundance in the wild-type strain and the Δ*shoA*, Δ*msbA*, and Δ*opyA* mutants when exposed to caspofungin 2 μg/ml for 1 h. (B) Venn diagrams comparing the abundances of proteins in the wild-type and each mutant strain. (C to F) A summary of the FunCat terms overrepresented with respect to increased or decreased abundance (adjusted *P* value of <0.05) in the wild-type strain exposed to caspofungin and compared to the control (C), in the Δ*shoA* mutant versus the wild-type strain posttransfer to caspofungin (D), in the Δ*msbA* mutant versus the wild-type strain posttransfer to caspofungin (E), and in the Δ*opyA* mutant versus the wild-type strain posttransfer to caspofungin (F). For the full list, refer to Table S1.

We used label-free quantitative proteomics (spectral counts) to investigate proteins that are differentially abundant in the Δ*shoA*, Δ*msbA*, and Δ*opyA* mutants upon exposure to caspofungin stress (Fig. 7; see also Table S1). We decided to expose the wild-type and the single mutants to caspofungin (8 μg/h for 1 h) because this concentration and this exposure time are important for CPE, and all the three single mutants lost the CPE (37) (Fig. 2K). Upon exposure to caspofungin (8 μg/h for 1 h), we observed increased abundances of 50, 109, 206, and 148 proteins and decreased abundances of 125, 130, 108, and 165 proteins in the wild-type, Δ*shoA*, Δ*msbA*, and Δ*opyA* strains, respectively (Fig. 7A). Venn diagrams were generated to correlate the protein abundance profiles for each strain treated with caspofungin (Fig. 7B). Although each strain had a unique set of proteins (135 for the wild-type strain, 94 for the Δ*shoA* mutant, 142 for the Δ*msbA* mutant, and 137 for the Δ*opyA* mutant), there was extensive overlap among the three mutant strains (Fig. 7B), suggesting independent and common roles for ShoA, MsbA, and OpyA in distinct pathways involved in the caspofungin response. Proteins of significant differential abundances in the Δ*shoA*, Δ*msbA*, and Δ*opyA* mutants were classified in terms of biological function. Upon caspofungin stress, in the wild-type strain there was an increase in the abundance of proteins involved in but not limited to (i) C-2 compound and organic acid metabolism and (ii) proteasomal degradation. There was also a decrease in the abundance of proteins involved in transcription and translation (Fig. 7C). In the mutant strains, there was an increase in the abundance of proteins involved in but not limited to (i) heat shock response, (ii) unfolded protein response, (iii) mitochondrial function, and (iv) protein folding and stabilization (Fig. 7D to F). In these strains, there was a reduction in the abundance of proteins belonging to categories such as (i) proteasomal degradation; (ii) sugar, glucoside, and polyol; (iii) stress response; (iv) glycolysis and gluconeogenesis; (v) pentose phosphate pathway; and (vi) C-compound and metabolism (Fig. 7D to F). Therefore, this proteomic analysis implies that upon caspofungin exposure, the absence of ShoA, MsbA, and OpyA affects the osmotic stress response, carbohydrate metabolism, and protein degradation.

### ShoA, MsbA, and OpyA are important for the utilization of several carbon sources.

**FIG 8:**
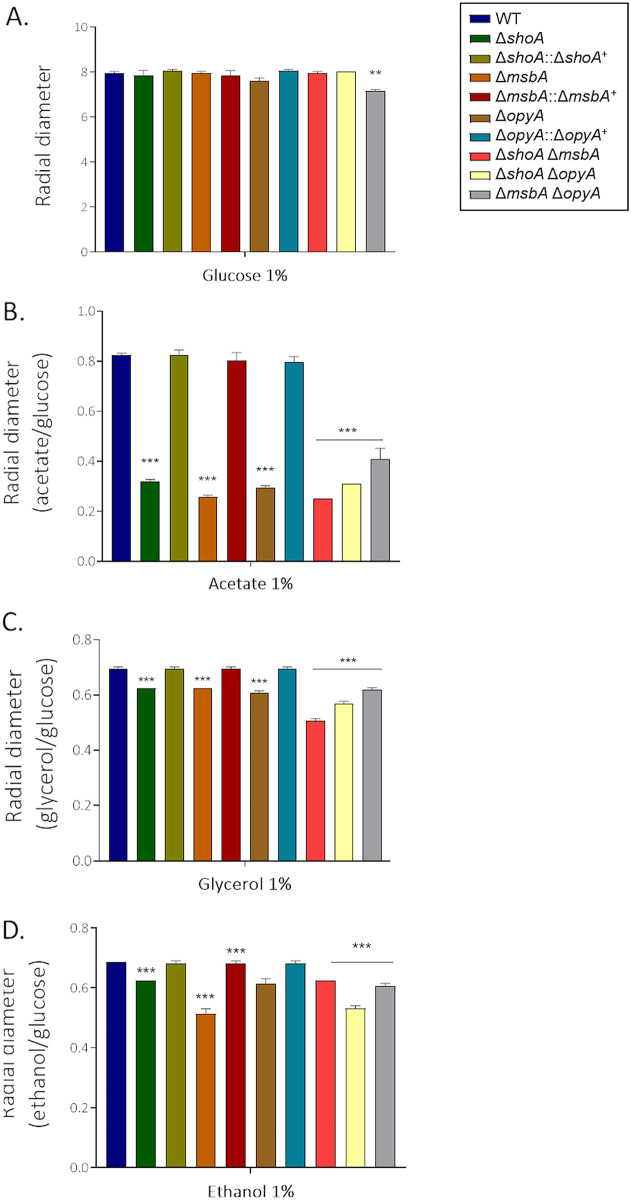
The Δ*shoA*, Δ*msbA*, and Δ*opyA* single and double null mutants showed reduced growth on glucose 1% (A), acetate 1% (B), glycerol 1% (C), or ethanol 1% (D) as a single carbon source. The wild-type and null mutant strains were grown for 5 days at 37°C. The results shown represent the means of measurements of the diameter of 3 colonies for each strain ± standard deviation. The data were analyzed (Prism, GraphPad) using two-way ANOVA followed by Bonferroni posttests. The levels of significance compared to the wild-type results are indicated as follows: *, *P* < 0.1; **, *P* < 0.01; ***, *P* < 0.001.

**FIG 9:**
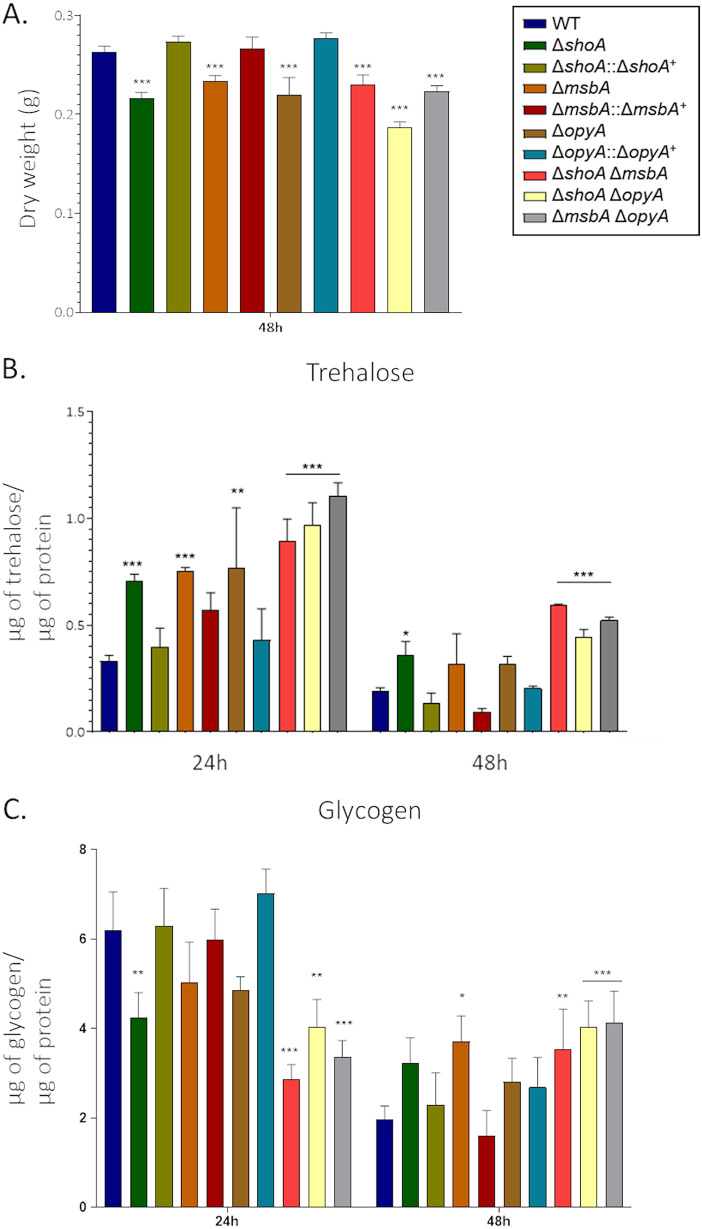
Altered trehalose and glycogen accumulation in the Δ*shoA*, Δ*msbA*, Δ*opyA*, and double null mutants. The strains were grown for 24 or 48 h in MM plus 1% glucose. The results shown represent the means of measurements of the diameter of 3 colonies for each strain ± standard deviation. (A) Dry weight. (B) Trehalose accumulation. (C) glycogen accumulation. The data were analyzed (Prism, GraphPad) using two-way ANOVA followed by Bonferroni posttests. The levels of significance compared to the wild-type results are indicated as follows: *, *P* < 0.1; **, *P* < 0.01; ***, *P* < 0.001.

Proteomic data from the Δ*shoA*, Δ*msbA*, and Δ*opyA* mutants revealed that many proteins involved in the catabolism of glucose and other sugars and amino acids, as well as in glucogenesis, were decreased in abundance (see Table S1). The single and double mutants showed about 10% to 15% growth reduction compared to the wild-type strain on 1% glucose (Fig. 8A), as well as reduced growth on 1% acetate, 1% glycerol, and 1% ethanol (Fig. 8B to D). For each carbon source (acetate, glycerol, or ethanol), the single and double mutants showed about the same level of growth reduction, suggesting that they function in the same pathway for assimilation of these carbon sources (Tables 1 and 2). There was also a reduction in the dry weight of the single and double mutants after 48 h in liquid MM plus 1% glucose (Fig. 9A). Glucose transport was not affected in the mutant strains (see Fig. S4), suggesting that there were no defects in glucose assimilation in these mutants. However, there was increased trehalose accumulation in the single and double mutants at 24 h or 48 h or at both time points (Fig. 9B). We also observed decreased glycogen accumulation in the Δ*shoA* mutant after 24 h of growth and increased glycogen accumulation in the Δ*msbA* mutant at 48 h of growth compared to the wild-type strain (Fig. 9C). Glycogen accumulation was decreased and increased in all three double mutants at 24 and 48 h of growth, respectively (Fig. 9C). These results suggest that glucose metabolism and sugar storage are affected in the putative receptor mutants.

**FIG 10:**
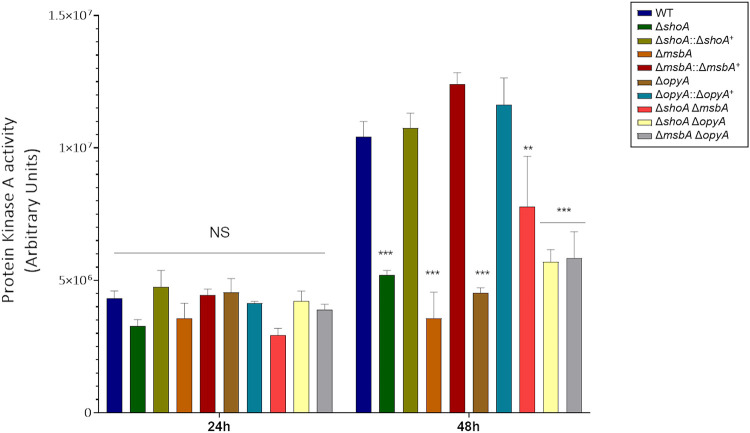
Protein kinase A activity was reduced in the Δ*shoA*, Δ*msbA*, Δ*opyA*, and double null mutants. The strains were grown for 24 or 48 h in MM plus 1% glucose. The results shown represent the means of measurements of three independent repetitions ± standard deviation. The data were analyzed (Prism, GraphPad) using two-way ANOVA followed by Bonferroni posttests. The levels of significance compared to the wild-type results are indicated as follows: *, *P* < 0.1; **, *P* < 0.01; ***, *P* < 0.001.

We recently observed that the MAPKs SakA and MpkC are involved in the A. fumigatus cell wall integrity pathway and interact with protein kinase A (PKA) to control carbohydrate mobilization for cell wall remodeling (38). Protein kinase A activity in the single and double mutants was comparable to that seen with the wild-type strain at 24 h of growth but was significantly reduced after 48 h of growth in glucose 1% (Fig. 10). Taken together, these results strongly indicate that the ShoA, MsbA, and OpyA putative receptors can affect carbon source utilization and protein kinase A activity.

### The Sln1 branch: genetic interactions between the two branches.

**FIG 11:**
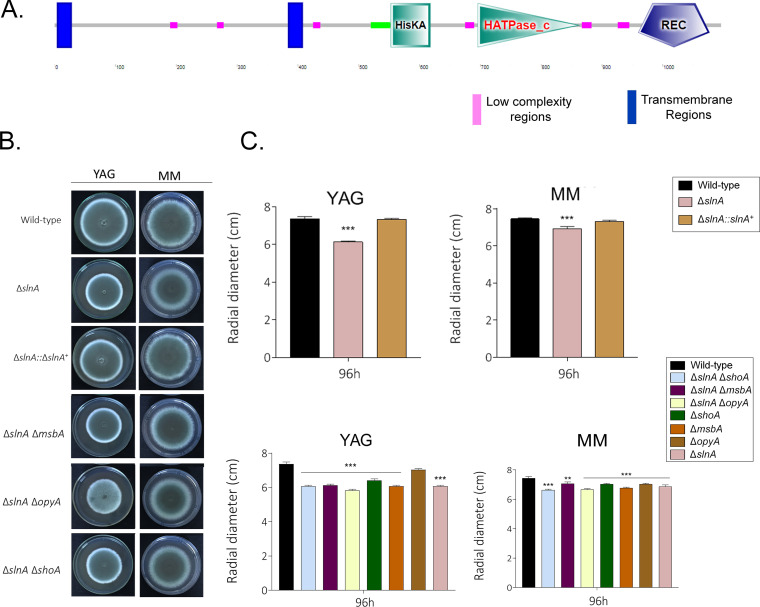

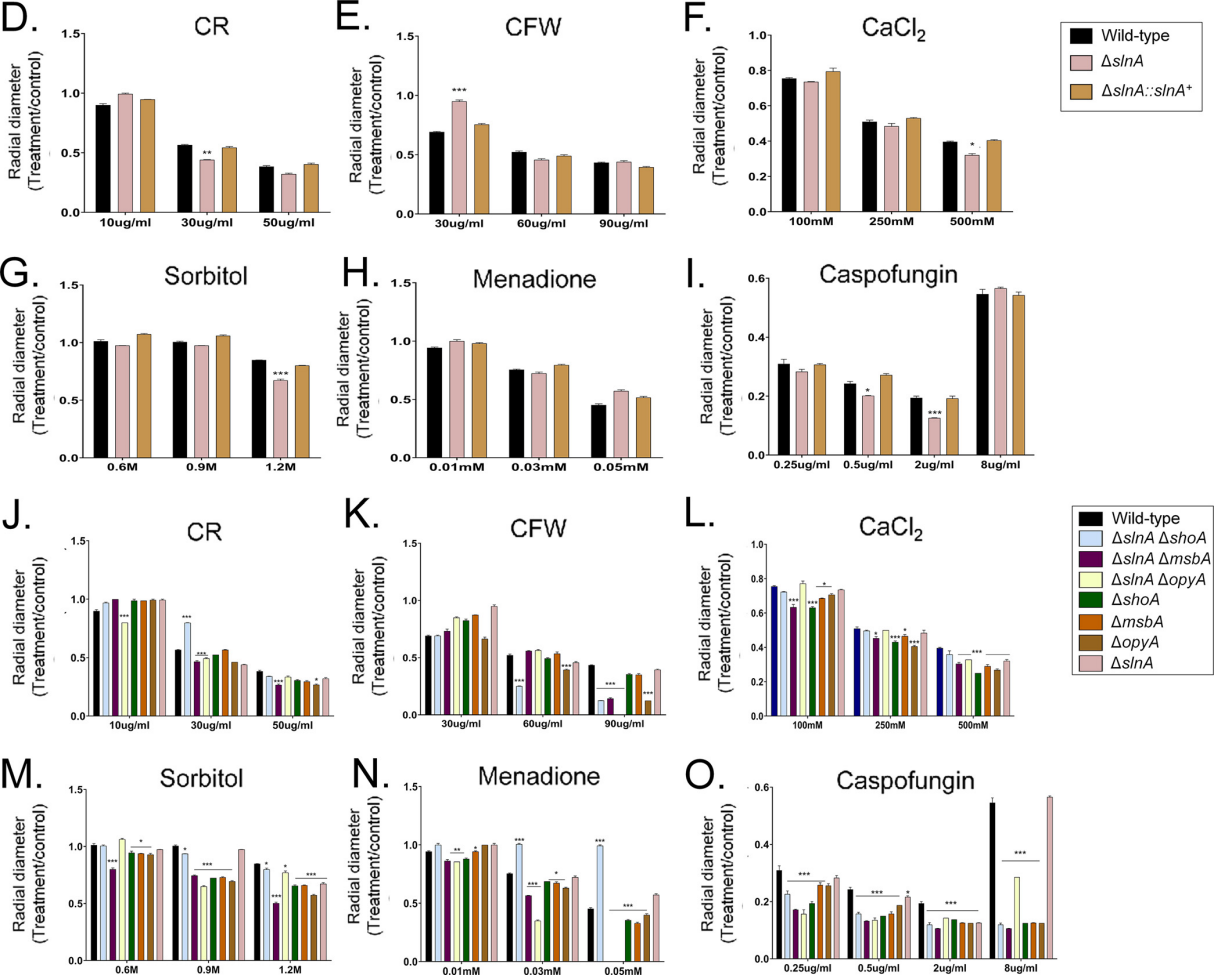
A. fumigatus SlnA and ShoA branches are genetically interacting. (A) Protein organization of A. fumigatus SlnA. (B to O) Growth phenotypes of the wild-type strain and the Δ*slnA*, Δ*shoA*, Δ*msbA*, Δ*opyA*, Δ*slnA* Δ*shoA*, Δ*slnA* Δ*msbA*, and Δ*slnA* Δ*opyA* mutants were determined. The strains were grown for 4 days at 37°C (B) on MM and YAG (C), MM plus Congo red (CR) (D and J), MM plus calcofluor white (CFW) (E and K), MM plus CaCl_2_ (F and L), MM plus sorbitol (G and M), MM plus menadione (H and N), and MM plus caspofungin (I and O), and their radial growth was quantified. The results shown represent the means of measurements of the diameter of 3 colonies for each strain ± standard deviation. The data were analyzed (Prism, GraphPad) using two-way ANOVA followed by Bonferroni posttests. The levels of significance compared to the wild-type results are indicated as follows: *, *P* < 0.1; **, *P* < 0.01; ***, *P* < 0.001.

An A. fumigatus Sln1p homologue was previously identified (22). We validated its organization as follows: SlnA^Sln1p^ (AFUA_2G00660, also called TcsB, www.aspgd.org) has 1,096 amino acids and 43% identity and 63% similarity with Sln1p (E value = 2e−40); it has two transmembrane regions, a HisKA domain (Interpro IPR003661) and a His kinase A phosphoacceptor domain (from amino acid 553 to amino acid 618); HATPase_c (Interpro IPR003594), a histidine kinase-like ATPse (from amino acid 696 to amino acid 863); and a REC domain (Interpro001789), a CheY-homologous receiver domain (from amino acid 960 to amino acid 1077) (Fig. 11A).

The Δ*slnA* mutant has about 15% reduced growth compared to the wild-type strain in YAG and MM (Fig. 11B and C). The Δ*slnA* mutant is more sensitive to sorbitol (1.2 M), CaCl_2_ (500 mM), and caspofungin (0.5 to 2.0 μg/ml) and more resistant to CFW (30 μg/ml) than the wild-type and complemented strains but not to CFW, CaCl_2_, and menadione (Fig. 11D to I); however, the Δ*slnA* mutant retained the CPE (Fig. 11I). In the G. mellonella model, infection by the wild‐type and Δ*slnA*::*slnA^+^* strains resulted in 100% mortality 8 and 9 days postinfection, respectively (see Fig. S5), while the Δ*slnA* mutant showed a 70% mortality rate 10 days postinfection, which was not statistically significantly different from the rates seen with the wild-type and complemented strains according to Mantel-Cox and Gehan-Breslow-Wilcoxon tests (*P* < 0.001; see Fig. S5). The double mutants Δ*slnA* Δ*shoA*, Δ*slnA* Δ*msbA*, and Δ*slnA* Δ*opyA* were as virulent as the single mutants Δ*shoA*, Δ*msbA*, and Δ*opyA*, suggesting there are no genetic interactions between *slnA* and *shoA*, *msbA*, and *opyA* for the establishment of virulence in A. fumigatus (Tables 1 and 2).

To investigate possible genetic interactions between the SlnA and ShoA branches, we constructed the double mutants Δ*slnA* Δ*shoA*, Δ*slnA* Δ*msbA*, and Δ*slnA* Δ*opyA*. They had about 10% to 15% reduced growth compared to the wild-type strain in YAG and MM (Fig. 11B and C). Only the double mutant Δ*slnA* Δ*msbA* was susceptible to 50 μg/ml CR, suggesting there were no genetic interactions with the single mutants in the presence of CR (Fig. 11J; see also Tables 1 and 2). In contrast, all of the double mutants were more sensitive to 90 μg/ml CFW than the corresponding single mutants, suggesting an additive interaction between *slnA* and *shoA*, *msbA*, and *opyA* (Fig. 11K; see also Tables 1 and 2). All the double mutants were as sensitive to 500 mM CaCl_2_ and 1.2 M sorbitol as the corresponding single mutants, suggesting that *slnA*, *shoA*, *msbA*, and *opyA* function in the same pathway for calcium and osmotic stress signaling (Fig. 11L and M; see also Tables 1 and 2). The Δ*slnA* Δ*shoA* mutant was less sensitive to 0.05 mM menadione than the corresponding single mutants, suggesting suppression interactions between *slnA* and *shoA*, while the *slnA* and *msbA* mutants and *slnA* and *opyA* mutants were more sensitive to menadione than the corresponding single mutants, suggesting an additive interaction (Fig. 11N; see also Tables 1 and 2). There were no genetic interactions between *slnA* and *shoA*, *msbA*, and *opyA*, since the Δ*slnA* double mutants were more resistant to lower and CPE caspofungin concentrations than the corresponding single mutants (Fig. 11O; see also Tables 1 and 2).

**FIG 12:**
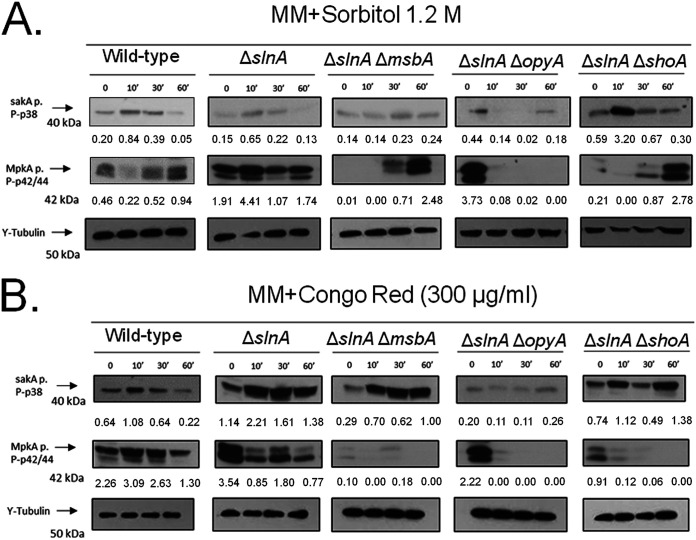
The A. fumigatus SlnA branch is important for SakA and MpkA phosphorylation. Western blotting assays of SakA and MpkA phosphorylation in response to 1.2 M sorbitol (A) and Congo red 300 μg/ml (B) were performed for different periods of time. Anti-P-p38 SakA and anti-44/42 MpkA antibodies were used to detect the phosphorylation of SakA and MpkA, respectively, while anti-γ-tubulin was used to detect γ-tubulin. Signal intensities were quantified using ImageJ software, and ratios of (P)-SakA to γ-tubulin or (P)-MpkA to γ-tubulin were calculated.

We also investigated the impact of *slnA* deletion on the phosphorylation levels of the MAP kinases SakA and MpkA in the presence of higher osmotic concentrations (1.2 M sorbitol) of CR instead of caspofungin as an alternative agent for mediating cell wall damage (Fig. 12; for an additional repetition of the Western blot assays, see Fig. S3). The wild-type strain showed increased SakA phosphorylation (about 4-fold) when exposed for 10 min to 1.2 M sorbitol (Fig. 12A). The Δ*slnA* mutant showed a level (about 4-fold at 10 min) of SakA phosphorylation comparable to that seen with the wild-type strain (Fig. 12A). In contrast, there was reduced SakA phosphorylation in the Δ*slnA* Δ*msbA* and Δ*slnA* Δ*opyA* strains whereas there was about a 5.4-fold induction of SakA phosphorylation at 10 min of exposure in the Δ*slnA* Δ*shoA* strain (Fig. 12A). There was about 2-fold- and 2.3-fold-increased MpkA phosphorylation in the wild-type and Δ*slnA* strains in the presence of 1.2 M sorbitol, while the Δ*slnA* Δ*msbA* strain showed increases of at least 7.1-fold and 24.8-fold in MpkA phosphorylation (Fig. 12A). The Δ*slnA* Δ*shoA* strain exposed to 1.2 M sorbitol for 30 and 60 min showed about 4.1-fold and 13.2-fold phosphorylation (Fig. 12A).

When exposed to CR (300 μg/ml), the wild-type strain showed about 1.7-fold and 1.4-fold more phosphorylation of SakA and MpkA, respectively, at 10 min (Fig. 12B). The increase in SakA phosphorylation in the Δ*slnA* mutant was comparable to that seen with the wild-type strain (about 1.9-fold) in the presence of CR, but no such increase in MpkA phosphorylation was seen (Fig. 12B). There were increases in SakA phosphorylation in the Δ*slnA* Δ*msbA* mutant of about 2.4-, 2.1-fold, and 3.4-fold in the presence of CR at 10, 30, and 60 min, respectively, and a late increase in SakA phosphorylation (about 1.9-fold at 60 min) in the Δ*slnA* Δ*shoA* mutant (Fig. 12B). There was no MpkA phosphorylation induction seen either in the Δ*slnA* single mutant or in the double mutants (Fig. 12B).

Taken together, these results strongly indicate that the SlnA and ShoA branches have genetic interactions that influence growth under several stressing conditions and are important for the proper activation of the SakA and MpkA cascade and the response to osmotic and cell wall stresses.

## DISCUSSION

The ability of pathogenic fungi to survive hostile environments inside and outside the host depends on their ability to respond quickly and robustly to stress (39). MAPK signaling pathways are highly conserved, promoting fungal adaptation to stress by activating the phosphorylation cascades of kinases, which in turn accumulate in the nucleus, triggering adequate cellular responses to different stimuli (40). The S. cerevisiae HOG pathway has already been widely characterized (41, 42). Sho1p and Sln1p are two independent upstream branches that activate the HOG pathway (11). Hkr1, Msb2, and Opy2 interact with Sho1, activating the Ste20-Ste11-Pbs2-Hog1 kinase cascade (9, 11, 43). The Sln1p branch is important for the regulation of hyperosmolarity and hypo-osmolarity responses (11).

Further studies are needed to understand how A. fumigatus activates and controls its HOG pathway. The main objective of this study was to characterize the A. fumigatus putative homologues of the different components of the yeast HOG pathway activation branches Sln1p and Sho1p. Accordingly, we characterized four putative genetic determinants involved in these two branches: SlnA^Sln1p^, ShoA^Sho1p^, MsbA^Msb2p^, and OpyA^Opy2p^. Apparently, there is no A. fumigatus Hrk1p homologue. We have shown that all four genes are not essential and that the Δ*slnA* mutant is not sensitive to any kind of investigated stress (except osmotic stress) or involved in virulence, while ShoA, MsbA, and OpyA are important for the cell wall integrity pathway and for oxidative stress and virulence. The construction and preliminary phenotypic characterization of Δ*shoA*, Δ*msbA*, and Δ*slnA* mutants were previously described (21–23). Most of the phenotypes described for the Δ*shoA* and Δ*msbA* mutants (21, 23) were also observed here. However, the growth of the Δ*slnA* mutant described here was more sensitive to osmotic stress than that of the Δ*slnA* strain previously described (22). Since the two studies used mutants derived from the same strain, differences could have been due to the growth conditions or medium composition.

The *shoA*, *msbA*, and *opyA* null mutants showed genetic interactions under stressing conditions, indicating that they interact for the activation of stress response mechanisms (Table 2). Although ShoA, MsbA, and OpyA have similar and/or redundant functions, they have unique functions during the activation of the signaling pathways in A. fumigatus. Interestingly, double mutants of the three genes in the Sho1p branch with Δ*slnA* show genetic interactions to different extents, strongly suggesting the two pathways interact in response to stress conditions. The modulation of MAPK HOG SakA and CWI MpkA phosphorylation levels is altered in these mutants, indicating that these two branches are important for activation of not only HOG but also CWI MAPKs. We cannot discard the possibility that these receptors also affect the MAPK phosphatases, altering the global levels of SakA and MpkA dephosphorylation. We made several unsuccessful attempts to construct triple null mutants with the three putative TM receptors. We were also unable to construct a triple mutant with a conditional *xylp*::*shoA* gene in the Δ*msbA* Δ*opyA* background under both repressing and inducing conditions. A single *xylp*::*shoA* mutant strain was not able to grow in xylose and induced high SakA and MpkA phosphorylation levels. These results provide direct evidence that the ShoA and SlnA branches are able to modulate activities of MAPKs.

In yeast, genetic analyses showed that the S. cerevisiae Sho1-Opy2 interaction enhances the activation of HOG Pbs2 MAPKKK by Fus3 MAPKK Ste11 but not the activation of Hog1 by Pbs2 (43). The interaction among these putative TM receptors has also been observed in many different human- and plant-pathogenic fungi. In the pathogenic fungus Candida albicans, *SLN1* deletion impaired hyphal formation and attenuated virulence (44). C. albicans *SHO1* mutants are also sensitive to oxidative stress and to CR and CFW and are defective in morphogenesis (45). C. albicans Msb2p is a global regulator of temperature stress (46), while *OPY2* mutants display susceptibility to CR, but it did not play a role in the adaptation to high osmolarity or oxidative stress and virulence in the Galleria mellonella model (47). Sho1 and Msb2 homologues play complementary but distinct roles in stress responses, differentiation, and pathogenicity of the human pathogen Cryptococcus neoformans (48). In the dimorphic plant-pathogenic fungus Ustilago maydis, Sho1 and Msb2-like proteins play a key role during appressorium differentiation and are involved in plant surface sensing (49). In the plant pathogen Verticillium dahliae, Sho1 acts as an osmosensor and is required for plant penetration and melanin biosynthesis (21). A Fusarium oxysporum Δ*msb2* mutant was shown to be essential for pathogenicity in tomato and hypersensitive to cell wall targeting compounds (50). F. oxysporum Δ*sho1* mutants were found to be hypersensitive to CFW, and a Δ*msb2* Δ*sho1* double mutant was even more sensitive. F. oxysporum Sho1 and Msb2 have converging functions activating the Fmk1 MAPK CWI cascade, promoting invasive growth and plant infection, and regulating the CWI pathway (51). These results emphasize the complex roles played by these putative TM receptors and Sln1p homologues in pathogenic fungi.

It is well documented that the response of A. fumigatus to caspofungin involves the combined action of HOG SakA and CWI MpkA MAPK (30, 37, 52). We investigated the protein spectral counts for the wild-type strain and the three putative TM receptor null mutants under unstressed conditions and when exposed to caspofungin. Our results revealed a completely novel aspect of the function of the putative TM receptors: their influence on growth on different carbon sources. The putative TM receptor null mutants showed reduced growth on several carbon sources compared to the wild-type strain. Although they do not have defects in glucose transport, they exhibit aberrant sugar storage metabolism, a strong indicator of problems in carbon assimilation metabolism. Cell wall-related sugars are derived from carbohydrate storage compounds during stress of the cell wall, and this process is dependent on cAMP-dependent protein kinase A (PKA) and on MAPKs SakA and MPkC (38). These protein kinases are important for normal accumulation/degradation of storage sugars through SakA physical interactions with the catalytic and regulatory subunits of protein kinase A PkaC1 and PkaR (38). The putative TM receptor mutants have reduced protein kinase A activity, and the effects on SakA phosphorylation could be the main reason for the reduced growth on several carbon sources.

In summary, we have provided novel information about A. fumigatus SlnA^sln1p^ and ShoA^Sho1p^ branches and their function in the activation of HOG MAPK pathway. Interestingly, these putative receptors also affect the CWI MpkA pathway. Phosphorylation of CWI MpkA MAPK in response to osmotic and cell wall stress was found to be dependent on HOG MpkC and SakA MAPKs (28, 33). These results indicated that there is a collaboration between the CWI and HOG pathways during cell wall biosynthesis (28, 33). Our work opens new possibilities for understanding the pathways for the activation and maintenance of osmolarity and the cell wall integrity in A. fumigatus. Those pathways are promising targets for drug development and could help to improve the efficacy of antifungal treatment and aspergillosis treatment.

## MATERIALS AND METHODS

### Strains and culture medium.

The strains were cultured at 37°C in complete yeast glucose (YG, 2% [wt/vol] glucose, 0.5% [wt/vol] yeast extract, trace elements) or minimal medium (MM; 1% [wt/vol] original salts with high nitrate content, trace elements, pH 6.5). For YAG and MM solid media, 1.7% (wt/vol) or 2% (wt/vol) agar were added to YG and MM. Where necessary, urine and uracil (1.2 g/liter) were added. The strains used in this work are described in Table S2 at https://figshare.com/articles/Membrane_receptors_contribute_to_activation_and_efficient_signaling_of_Mitogen-Activated_Protein_Kinase_cascades_during_adaptation_of_Aspergillus_fumigatus_to_different_stressors_and_carbon_sources/12402125.

### Construction of A. fumigatus null mutants.

A. fumigatus strain CEA17 was used to generate the following deletion strains used in this study: the Δ*msbA*, Δ*opyA*, and Δ*shoA* single mutants, with sequences corresponding to *msbA* (Afu4g04070), *opyA* (Afu3g14410), and *shoA* (Afu2g00660) according to the AspGD database (http://www.aspgd.org/). The deletion cassettes were constructed by *in vivo* recombination in S. cerevisiae as described previously (53). We used approximately 1.0 kb of the 5′ untranslated region (5′-UTR) and the 3′-UTR flanking the target open reading frame (ORF) regions was selected for primer design (see Table S3). Primers 5'Fw and 3'Rv contained a short sequence homologous to plasmid pRS426 and the *pyrG* gene. Both the 5′-UTR and 3′-UTR fragments were amplified by PCR from A. fumigatus genomic DNA (gDNA). The *pyrG* gene placed inside the cassette as a prototrophic marker was amplified from plasmid pCDA21. The deletion cassette was generated by transformation into S. cerevisiae SC94721 using the lithium acetate method (54), plus the fragments, together with plasmid pRS426 digested at two sites with enzymes BamHI and EcoRI (New England Biolabs Ltd., United Kingdom). The DNA of the transformants was extracted by a previously described method (55). The deletion cassettes were amplified by PCR from these plasmids using TaKaRa *Ex Taq* DNA polymerase (Clontech TaKaRa Bio) and were used for the transformation of A. fumigatus. The same protocol was used for the construction of the double mutants. However, the marker gene used for the deletion of the gene was amplified from plasmid pPTRI. The colonies of null mutants were selected by purification of the colonies in 1 μg/ml pyrithiamine hydrobromide (Sigma). The results of Southern blot analysis demonstrated that the transformation cassette was homologously integrated (see Fig. S1). Single gene deletion strains were complemented by cotransformation of approximately 1.0 kb of the 5′-UTR and 3′-UTR plus ORF together with plasmid pPTRI. The complementants were selected by purification of colonies in MM with 1 μg/ml pyrithiamine hydrobromide. Gene complementation was checked by PCR (see Fig. S1). All the primers used in this work are described in Table S3.

### Construction of *xylP*::*shoA* strain.

The strain of A. fumigatus (CEA17) with *pyrG* auxotrophic mutant marker was used to generate strain *xylP*::*shoA*, whose *shoA* gene was fused to the *xylP* promoter, activated by xylose. The cassettes were constructed by *in vivo* recombination in S. cerevisiae (53), and we used about 1.0 kb of the 5′-UTR flanking region of the region and 3′-UTR of the target ORF regions were selected for the design of the primer. The *xylP* promoter was amplified from the plasmid pYES-pXyl-hph-devR. The *pyrG* gene placed inside the cassette as a prototrophic marker was amplified from plasmid pOB430. The fragments necessary for the construction of the cassette were amplified by PCR using the primers listed in Table S3. The construction of the cassette was performed *in vivo* using S. cerevisiae SC9721 and the lithium acetate method (54), plus the fragments, together with plasmid pRS426 digested in two sites with the enzymes BamHI and EcoRI (New England Biolabs Ltd., United Kingdom). The DNA of the transformants was extracted using a previously described method (55). The *xylP*::*shoA* construct cassette was amplified by PCR from these plasmids using TaKaRa *Ex Taq* DNA polymerase (Clontech TaKaRa Bio) and used for the transformation of A. fumigatus. The colonies of the transformants were selected by purification in MM (1% glucose). Candidates were checked by PCR using 5′-UTR primers external to the cassette used for transformation (see Fig. S1).

### Characterization of mutants.

Sensitivity and resistance to several stressing agents were evaluated by measurement of radial growth, comparing the mutant and wild-type strains. A 5-μl volume of a suspension of 2 × 10^7^ conidia was inoculated into MM with or without a corresponding stressing agent, and the plates were incubated for 4 days at 37°C. After this period, the radial diameters were measured and the values were used for statistical analysis. The data were analyzed (Prism, GraphPad) using two-way analysis of variance (ANOVA) followed by Bonferroni posttests. The levels of significance compared to the wild-type results are indicated as follows: *, *P* < 0.1; **, *P* < 0.01; ***, *P* < 0.001.

### Immunoblot analysis.

The strains were inoculated (1 × 10^7^ conidia) in 250-ml Erlenmeyer flasks with 50 ml of YAG medium and grown in a rotatory shaker (200 rpm) at 37°C for 16 h before being exposed to different stressing agents, such as Congo red, hydrogen peroxide, sorbitol, and calcium chloride. The proteins were extracted as described previously (56) and quantified according to a previously described method (57). A 60-μg volume of total protein per sample was run on a 12% (wt/vol) SDS-PAGE gel before being transferred to a polyvinylidene difluoride (PVDF) membrane (GE Healthcare). The phosphorylated forms of MpkA and SakA were detected using anti-phospho-p44/42 MAPK and anti-phospho-p38 MAPK (Cell Signaling Technology) antibodies, respectively. Primary antibodies were detected using an anti-rabbit IgG antibody, horseradish peroxidase (HRP) antibody (catalog no. 7074; Cell Signaling Technologies). The detection (chemiluminescence) was performed using a Western ECL Prime (GE Healthcare) blot detection kit according to the manufacturer's instructions. The phosphorylated signal of MpkA and SakA was normalized to the monoclonal antibody IgG γ-tubulin (C-11, sc-17787) ratio. The images generated were submitted for densitometric analysis using ImageJ software (http://rsbweb.nih.gov/ij/index.html).

### Enzymatic assays.

Analyses of intracellular trehalose levels (Megazyme) and PKA activity (Promega) were carried out according to the manufacturer’s instructions. Intracellular glycogen levels and extracellular glucose concentrations were quantified as described previously (58).

### Cell wall staining.

Cell wall surface polysaccharide straining was performed as described previously (28, 59, 60). Briefly, strains were grown from 2.5 × 10^3^ spores in 200 μl of MM for 16 h at 37°C before the culture medium was removed and germlings were UV irradiated. The hyphal forms were subsequently washed with PBS 1×, and 200 μl of a blocking solution (2% [wt/vol] goat serum, 1% [wt/vol] bovine serum albumin [BSA], 0.1% [vol/vol] Triton X-100, 0.05% [vol/vol] Tween 20, 0.05% [vol/vol] sodium azide, and 0.01 M PBS) was added. Samples were incubated for 30 min at room temperature. For dectin staining, 0.2 μg/ml of Fc-h-dectin-hFc was added to the UV-irradiated germlings and incubated for 1 h at room temperature, followed by the addition of 1:1,000 DyLight 594-conjugated, goat anti-human IgG1 for 1 h at room temperature. The hyphal forms were washed with PBS, and fluorescence was read at 587 nm excitation and 615 nm emission. For chitin staining, 200 μl of a PBS solution with 10 μg/ml of calcofluor white (CFW) was added to the UV-irradiated hyphal forms, incubated for 5 min at room temperature, and washed three times with PBS before fluorescence was read at 380 nm excitation and 450 nm emission. All experiments were performed using 12 repetitions, and fluorescence was read in a microtiter plate reader (SpectraMax i3; Molecular Devices).

### Virulence analysis in Galleria mellonella models.

The Galleria mellonella larvae were obtained by breeding adult larvae (61) weighing 275 to 330 mg, kept starving in petri dishes at 37°C in the dark for 24 h prior to infection. All selected larvae were in the final (sixth) stage of larval development. Fresh conidia of each strain of A. fumigatus were counted using a hemocytometer, and the initial concentration of the suspensions of conidia used for the infections was 2 × 10^8^ conidia/ml. A total of 5 μl (1 × 10^6^ conidia/larva) of each suspension was inoculated per larva. The control group was composed of larvae inoculated with 5 μl of PBS to observe death by physical trauma. The inoculation through the last left proleg was performed using a Hamilton syringe (model 7000.5 KH). After infection, the larvae were kept at 37°C in petri dishes in the dark and scored daily. Larvae were considered dead due to lack of movement in response to touch. The viability of the inoculum administered was determined by plating a serial dilution of the conidia on YAG medium and incubating the plates at 37°C for 72 h. All the experiments were performed in parallel and repeated twice.

### Purification and identification of proteins by LC-MS/MS.

To precipitate crude protein extracts, they were prepared from Δ*shoA*, Δ*opyA*, and Δ*msbA* mutant and wild-type cultures that were grown for 24 h and exposed to caspofungin 8 μg/ml (60 min). Crude mycelium protein extracts were obtained by extraction of ground mycelium by the use of B250 buffer (250 mM NaCl, 100 mM Tris-HCl [pH 7.5], 10% glycerol, 1 mM EDTA, and 0.1% NP-40) supplemented with 1.5 ml/liter 1 M dithiothreitol (DTT), 2 tablets/100 ml complete EDTA-free mini-protease inhibitor cocktail (Roche), 3 ml/liter 0.5 M benzamidine, 10 ml/liter phosphatase inhibitors 100× (10 M NaF, 5 M sodium vanadate, 8 M β-glycerol phosphate), and 10 ml/liter of 100 mM phenylmethylsulfonyl fluoride (PMSF). Total protein lysates were centrifuged at 13,000 rpm at 4°C for 10 min, and the supernatant was collected in a new Eppendorf tube. Samples were quantified by the Bradford method (62) and subjected to trypsinization by the use of trypsin (Promega; catalog no. 9PIV511) according to the manufacturer’s instructions. The peptides obtained after trypsinization were bound to a resin (C18 ZipTip pipette tips) according to the manufacturer's instructions (Merck). Digested peptides were separated using reverse-phase liquid chromatography with an Ultimate 3000 NanoLC system (Dionex Corporation, Sunnyvale, CA, USA) followed by mass identification using a Q Exactive mass spectrometer (Thermo Fisher Scientific). Samples were loaded by the use of an autosampler onto a C_18_ trap column, which was switched on-line with an analytical BioBasic C_18_ PicoFrit column (C_18_ PepMap; Dionex) (75-μm inside diameter [i.d.] by 500 mm, 2 μm particle size, 100 Å pore size). Full scans in 300 to 1,700 *m*/*z* were recorded with a resolution of 140.000 (*m*/*z* 200) with a blocking mass set to 445.12003. Protein identification and label-free quantification (LFQ) normalization were performed using MaxQuant v1.5.2.8 quantitative proteomic software (www.maxquant.org/) in conjunction with Perseus v.1.5.6.0 statistical analysis software (www.maxquant.org/). ANOVA-based multisample *t* tests were performed using a *P* value cutoff point of <0.05, allowing the identification of differentially abundant, statistically significant proteins.

### RNA extraction and gene expression analysis.

Wild-type and *xylP*::*shoA* strains were inoculated (1 × 10^7^ conidia) in 50 ml of MM for 24 h at 37°C. Mycelia were transferred to MM plus 1% xylose for 30 to 240 min. Mycelia were ground to a fine powder in liquid N_2_, and total RNA was extracted with TRIzol reagent (Thermo Scientific) according to the manufacturer’s protocol. DNA was digested with Turbo DNase I (Ambion Thermo Scientific) according to manufacturer’s instructions. A 2-μg volume of total RNA per sample was subjected to reverse transcription with a High Capacity cDNA reverse transcription kit (Thermo Scientific) using a blend of oligo(dTV) and random primers, according to the manufacturer’s instructions. Quantitative real-time PCRs (qRT-PCRs) were run in a StepOne Plus real-time RT-PCR system (Thermo Scientific) using Power Sybr green PCR master mix (Thermo Scientific). Three independent biological replicates were used, and the mRNA quantity relative fold change values were calculated using standard curves (63). All values were normalized to the expression of the A. fumigatus *tubA* gene. The primers are described in Table S3.

### Data availability.

The proteomic data set can be accessed in Table S1 at https://figshare.com/articles/Membrane_receptors_contribute_to_activation_and_efficient_signaling_of_Mitogen-Activated_Protein_Kinase_cascades_during_adaptation_of_Aspergillus_fumigatus_to_different_stressors_and_carbon_sources/12402125.
